# The Effect of Multiple Paternity on Genetic Diversity of Small Populations during and after Colonisation

**DOI:** 10.1371/journal.pone.0075587

**Published:** 2013-10-28

**Authors:** Marina Rafajlović, Anders Eriksson, Anna Rimark, Sara Hintz-Saltin, Grégory Charrier, Marina Panova, Carl André, Kerstin Johannesson, Bernhard Mehlig

**Affiliations:** 1 Department of Physics, University of Gothenburg, Gothenburg, Sweden; 2 Department of Zoology, University of Cambridge, Cambridge, United Kingdom; 3 Integrative Systems Biology Lab, King Abdullah University of Science and Technology, Thuwal, Kingdom of Saudi Arabia; 4 Department of Biological and Environmental Sciences-Tjärnö, University of Gothenburg, Strömstad, Sweden; Biodiversity Insitute of Ontario - University of Guelph, Canada

## Abstract

Genetic variation within and among populations is influenced by the genetic content of the founders and the migrants following establishment. This is particularly true if populations are small, migration rate low and habitats arranged in a stepping-stone fashion. Under these circumstances the level of multiple paternity is critical since multiply mated females bring more genetic variation into founder groups than single mated females. One such example is the marine snail *Littorina saxatilis* that during postglacial times has invaded mainland refuge areas and thereafter small islands emerging due to isostatic uplift by occasional rafting of multiply mated females. We modelled effects of varying degrees of multiple paternity on the genetic variation of island populations colonised by the founders spreading from the mainland, by quantifying the population heterozygosity during both the transient colonisation process, and after a steady state (with migration) has been reached. During colonisation, multiple mating by 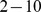 males increased the heterozygosity by 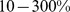 in comparison with single paternity, while in the steady state the increase was 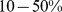 compared with single paternity. In the steady state the increase of heterozygosity due to multiple paternity is determined by a corresponding increase in effective population size. During colonisation, by contrast, the increase in heterozygosity is larger and it cannot be explained in terms of the effective population size alone. During the steady-state phase bursts of high genetic variation spread through the system, and far from the mainland this led to short periods of high diversity separated by long periods of low diversity. The size of these fluctuations was boosted by multiple paternity. We conclude that following glacial periods of extirpation, recolonization of isolated habitats by this species has been supported by its high level of multiple paternity.

## Introduction

When local populations are established or re-established after local extirpation, genetic variation within the newly founded populations is initially governed by the genetic content of the founders, which, in the most extreme case, may be a single fertilised female. If new populations are established in a stepping-stone fashion at increasing distances from a large source population, the genetic content of founder groups is even more important, since loss of genetic variation by drift is expected to increase as the number of colonisation steps becomes larger. At a later stage, during continued input of variation through migration, the genetic composition of migrants may in a similar way contribute new variation and hence counteract loss by drift and selection.

Furthermore, the genetic variation carried by the founders is expected to be influenced by multiple paternity in brooding and sexual species. Females mating multiple males usually have broods of offspring sired by more than one male, although sperm competition or cryptic female choice may cause deviations from a random distribution of paternity [1]. Multiple paternity, once believed to be rare in nature, is observed in a number of animal species including mammals, amphibians, fishes, reptiles and invertebrates [2]–[5]. In most species levels of multiple paternity are around two to four males, but in some species of fish and invertebrates it is rather six to ten [6]–[9]. The marine snail *Littorina saxatilis* is an extreme example with 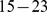 males siring broods of single females [10].

**Figure 1:**
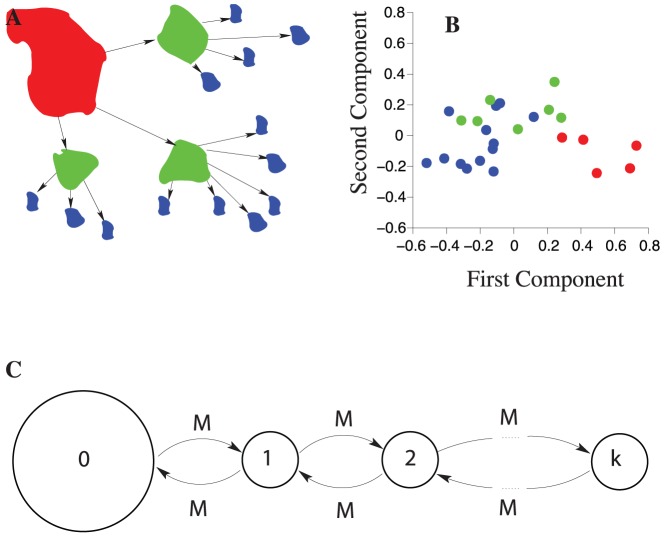
Spatial structure of the marine habitats of *Littorina saxatilis*. (**A**) A schematic illustration of the geographical structure of populations with mainland (red), islands (green), and skerries (blue). (**B**) Principal components of allozyme population differentiation in *L. saxatilis* (data from [13], the presumably selected locus 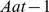 is excluded). Populations are classified as mainlands (red), islands (green), or skerries (blue). (**C**) Stepping-stone model of a section of the archipelago, with the mainland (labelled by 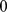) acting as a source for establishing the island populations (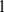 to 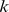). On average, each island sends 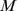 females per generation to its right and to its left neighbouring island (but, note that the mainland and the island furthest from the mainland have only one neighbour).

In the near future, whole-genome sequences will become available for many species. In order to be able to deduce the factors driving the long-term evolution of natural populations from genome-wide patterns of genetic variation, it is necessary to quantify the effects of life history (e. g. mating patterns, multiple paternity) and geography (e. g. population structuring). The aim of this paper is to quantitatively understand how multiple paternity and geographic structure in the form of a stepping-stone model determine patterns of neutral genetic variation. The marine snail *Littorina saxatilis* is an example for which a stepping-stone colonisation model with a mainland as a source population describes the establishment of new populations. This species is strictly intertidal and most abundant in rocky shores in the north Atlantic, with population densities of tens to hundreds of snails per square metre [11]. In contrast to many other marine snails, *L. saxatilis* does not have pelagic eggs or larvae, and therefore dispersal over a few tens of metres range is infrequent. However, snails occasionally migrate among islands, probably by rafting. It has been estimated that within an archipelago of small and large islands, 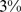 of the small islands receive a migrant snail each year [12]. In many areas, *L. saxatilis* forms local populations inhabiting discrete localities, such as islands of an archipelago, rocky outcrops and breakwaters intermingled by sandy substrates [13], [14]. During the retreat of the ice sheet 12 000–15 000 years ago, *L. saxatilis* spread from refuge areas both in the northern Atlantic and south of the ice-cap [10]. Part of this postglacial expansion comprised colonisation of hundreds of islands in the archipelago along the Swedish west coast that successively became available by isostatic uplift, a process that is still ongoing. In this system, populations on the mainland and large islands are the oldest and largest, and these are likely to act as the ultimate sources of genetic variation during colonisation of emerging islands in a stepwise manner (Fig. 1 **A**). We have re-analysed genetic data from *L. saxatilis* populations in the archipelago of west Sweden (given in Table 1 in [13]) using a principal component analysis. The data consist of allelic frequencies at four polymorphic allozymes (we excluded the locus 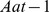) taken at thirteen skerries (skerry sizes 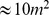), seven islands (island sizes 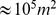), and five mainlands. We have found that the first principal component shows a largely linear relationship between population-genetic variation and size/age of the islands/populations, with mainland populations at the one end, skerry populations at the other end, and island populations in the middle (Fig. 1 **B**). This, together with the findings of [12] (see above), suggests that a section of the archipelago consisting of the neighbouring mainland-, island- and skerry-population can be modelled by a simple linear stepping-stone model with the mainland population acting as a source for colonisation of islands at successively younger age, and at increasing distance from the mainland (Fig. 1 **C**).

An important well-known characteristic of *L. saxatilis* is multiple paternity [10]. In the following, we investigate how multiple paternity affects the spatial and temporal structure of neutral genetic diversity of subpopulations arranged in a linear stepping-stone model. With the extreme level of multiple paternity of the marine snail in mind, we construct a mating model for this species and analyse how multiple paternity affects population-genetic variation and structure in a population distributed over both mainland sites and adjacent small island habitats. In our analysis, we consider established local populations in a global steady state, but also populations under establishment (during initial colonisation of previously empty habitats). We first derive an expression for the effective genetic population size [15] resulting from the mating behaviour observed in earlier empirical studies. This further allows us to derive simple approximations describing the genetic diversity of subpopulations during colonisation, and in the steady state with migration. We use simulations to assess the temporal effects of migration on genetic diversity as new mutations from the mainland spread to distant islands.

## Description of the Model

### Stepping-stone colonisation model

We construct a stepping-stone colonisation model with the following basic assumptions mimicking how *L. saxatilis* colonised the post-glacial archipelago of western Sweden (and continues to colonise new emerging islands). First, colonisation was occasional and rapid, as rafted fertilised females release a few hundred offspring already in the first generation [12]. Second, small skerries are likely to be colonised within a few years after emergence, and hence all newly established populations are limited by the small size of the habitat, resulting in census sizes of 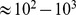 [12]. Third, colonisation is likely to take place in a stepping-stone manner with smaller and more distantly related islands being colonised from their closest already colonised islands. For simplicity, we consider a system consisting of a mainland and of linearly arranged islands of equal carrying capacities (substantially smaller than that of the mainland).

In our model, islands are linearly arranged and numbered from 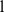 to 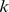, with 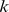 being furthest away from the mainland (see Fig. 1 **C**). We include high values of 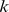 in our model (such as 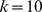) in order to be able to assess saturation effects. The mainland is labelled by 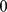. Generations are assumed to be non-overlapping. Before the process of colonisation starts, the mainland is the only populated habitat, and the population heterozygosity on the mainland is stationary (that is, the mainland population is assumed to be old). We assume that mutations accumulate according to the infinite-alleles model [16].

Within our model, an empty island becomes fully colonised in a single generation after the arrival of one or more founder females from the nearest neighbouring island. This is motivated by the very large capacity for population growth of *L. saxatilis* in a suitable habitat [12], [13]. In our model the founder females give rise to 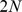 offspring in total, with equal sex ratio, where 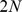 denotes the carrying capacity of the island. Upon establishing a given island population, its population size remains constant over time. In our model, mating takes place before migration, which allows us to trace only the movement of females (males also migrate, but since they will not mate after migration, they do not contribute to the progeny on the island they migrated to). Individuals are equally likely to migrate to each of their closest neighbours (but the mainland and the last island have only a single neighbour, Fig. 1 **C**). On average, 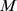 females migrate per generation from one island to a neighbouring island, except for empty islands that only receive migrants.

In addition to the above, we assume that the population size on the mainland is much larger than the population size of a colonised island. In our computer simulations we set the mainland heterozygosity to unity. This simplifies the simulations, since the dynamics of the mainland does not need to be simulated explicitly.

An important aspect of our model is the mainland, providing a source of genetic variation through repeated founder events. We remark that our model differs essentially from the mainland-island model analysed in [17] (see also references cited in this work). In [17] it is assumed that all island populations (called sink populations in [17]) either receive migrants from a source simultaneously, or are equally likely to receive migrants. In this case the degree of genetic variation in the steady state turns out to be the same for all islands. This is also true for stepping-stone models without a mainland (see for example Refs. [18], [19]).

### Mating model

In order to study the consequences of multiple paternity for genetic diversity, we introduce a mating model to describe different levels of multiple paternity in mating systems.

Based on known life-history traits [13], we assume that the duration of the reproductive cycle of females is the same for all females. Each female carries beneath her shell juveniles of varying degrees of maturity, and juveniles are released at an approximately steady rate. We also assume that after a successful mating, the mated female obtains a sperm package able to fertilise female eggs during its persistence time. Our observations show that sperm can be stored and used up to a year after mating. The number of eggs fertilised by a single sperm package is assumed to be the same for all sperm packages that a female receives during the reproductive cycle. The total number of active sperm packages received by each female during the reproductive cycle is denoted by 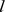, and 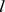 is assumed not to depend on time (it has the same value in every generation).

The probability 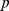 that two eggs are fertilised by the same sperm package is 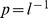 assuming that all sperm packages a female received during her reproductive cycle persist until the end of the reproductive cycle, that all eggs are fertilised after all sperm packages have been collected, and that sperm packages are chosen with replacement to fertilise eggs.

The scheme presented above models the process of mating at an individual level. We assume that individuals belong to a well mixed diploid population of 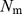 males and 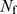 females, and we take 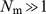 and 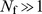. In our model, each female encounters 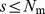 different males during the reproductive cycle (thus, 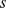 is assumed to be the same for all females, and it does not depend on time). Having 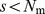 reflects the limited movement of snails during the reproductive cycle. Unlike under random mating, where all males are treated as on average equally successful mates, it is possible that females exhibit cryptic choice, or that some of the available mates of each female are more dominant than others. We include this in our model by assigning different levels of dominance to the available mates: we assume that one of the 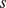 males (randomly chosen) has on average a higher success in mating, whereas the remaining 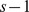 males have lower success. The dominant male mates the female with probability 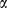, whereas the remaining 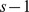 males mate the female with probability 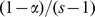. We also assume that all females are on average equally successful mothers.

In our model the probability that two offspring come from the same female, 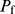, is given by
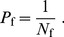
(1)Note that this implies that the brood sizes of females are multinomially distributed, similarly to the commonly used Wright-Fisher population [20], [21]. Furthermore, the probability that two offspring come from the same male, 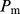, is
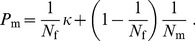
(2)In Eq. (2), the first term corresponds to the probability that two offspring share both a mother and a father, and the second term is the probability that two offspring come from different mothers but they share a father. The factor 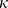 stands for the probability that two offspring having the same mother share a father, and it is given by
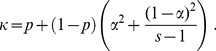
(3)Here, the first term is the probability that two eggs are fertilised by the same sperm package, and the second term stands for the probability that two eggs are fertilised by two distinct sperm packages, both coming from the same male. The probability that two packages come from the same male has two contributions. The first contribution, 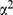, stands for the probability that both packages come from the dominant male, and the second contribution, 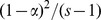, is the probability that two packages come from one of the remaining 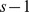 males. Note that, according to Eq. (3), 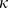 decreases as the number of available mates, 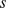, of a female increases (keeping the values of 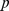 and of 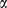 fixed). Therefore, we take 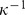 as a measure of the degree of multiple paternity.

Using Eqs. (1)–(3) we find that the effective size of a freely mixing population under the model described is given by:
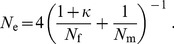
(4)The mathematical details of this derivation are given in **Appendix S1**. Our model reduces to the model in [22] in the case a female encounters all males present in the population, 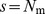, upon assuming that all males are on average equally successful mates, 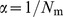, and upon identifying the number of matings in [22] with 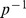. Furthermore, if 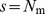, 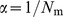, and the probability that two eggs are fertilised by the same sperm package is 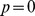, our model reduces to random mating. We also note that in two particular cases, our model reduces to two mating models analysed in [23]. First, for 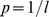, 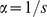, and 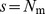 (or 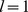), our model is analogous to the model of lottery polygyny in [23] in the case all females mate with certainty, and assuming non-overlapping generations (that is, upon setting 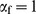, and 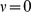 in [23]). Second, for 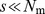, 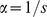, and 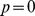, our model reduces to the model of dominance polygyny in [23] in the case of non-overlapping generations, and upon assuming that all males are dominant in this model (that is, for 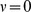, and 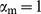 in [23]).

**Figure 2:**
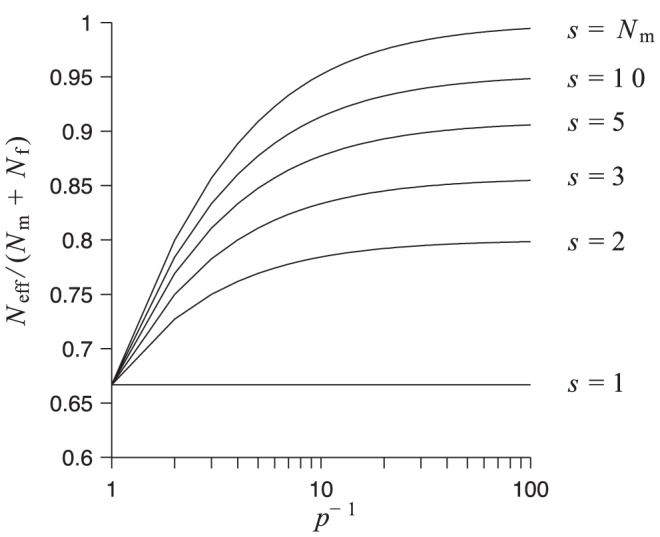
Effective population size. For different number of available mates, 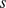, and probability that two eggs are fertilised by the same sperm package, 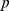, we show the ratio of the effective population size (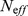) to the total population size (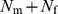). Parameters: 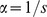, 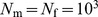.

We show in Fig. 2 how the effective population size under our model depends upon 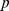 and 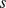. As expected, by increasing the degree of multiple paternity, the effective population size becomes larger (as found in [24] for a different mating model). For the parameters set in Fig. 2, the maximum value of 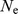 is equal to 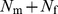, which corresponds to the effective population size under random mating. The increasing trend of 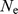 saturates at 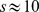 for a given value of 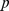 and 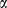.

We used empirical data to test our mating model from two experiments. In one experiment, six virgin females of similar sizes were placed in separate aquaria, and each was accompanied by ten equally sized males. The snails were allowed to mate freely during eight weeks, after which the males were removed, and offspring were collected from each female over a one-year period following the mating period. The sire of each offspring was determined by genotyping the males, females and juveniles at eight microsatellite loci and performing parentage analysis following an exclusion method (Saltin, S. H. et *al*, unpublished data). Only the offspring that could uniquely be assigned to a given sire were kept. Hence, 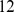 out of 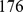 offspring were removed from the analysis.

In the second experiment, genotype data from five microsatellite loci were collected in four wild females and their offspring [10]. The brood sizes of the four wild females were 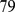, 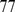, 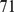, and 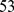 [10]. Since the potential sires were unknown, COLONY [25] was used to estimate the sibships of each female's offspring. In order to test the goodness of our model, we fitted our mating model to the distribution of male family sizes in the empirical data, using a chi-square test to assess goodness of fit (details of binning and degrees of freedom are given in the results section). Because the circumstances in the controlled mating experiments differ from that of females mated in the wild, we fitted the two datasets separately.

The brood sizes of the six females mated under experimental conditions in the lab was 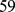, 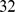, 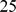, 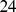, 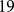, and 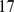 (Saltin, S. H. et *al*, unpublished data). For the offspring of each female it was determined how many came from the same father, that is, male family sizes. For the snails mated in the lab, the maximum male family size was 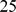 (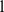 male). Remaining male families were of sizes 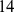 (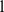 male), 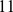 (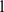 male), 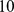 (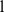 male), 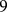 (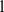 male), 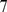 (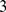 males), 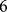 (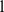 male), 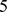 (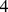 males), 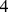 (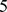 males), 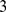 (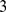 males), 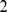 (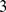 males), and 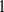 (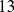 males). To avoid ambiguity in paternity, 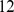 offspring were excluded from male family assignment, yielding broods of sizes 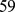, 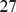, 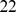 (two broods), 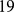, and 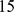 for further analysis. Similarly, for the snails mated in the wild, the male families were of sizes 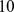 (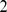 males), 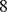 (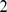 males), 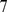 (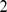 males), 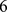 (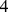 males), 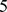 (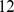 males), 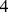 (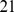 males), 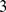 (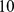 males), 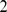 (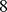 males), and 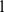 (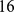 males). For the details on parental assignment in this case, see [10].

## Results

### Distribution of male family sizes

**Figure 3:**
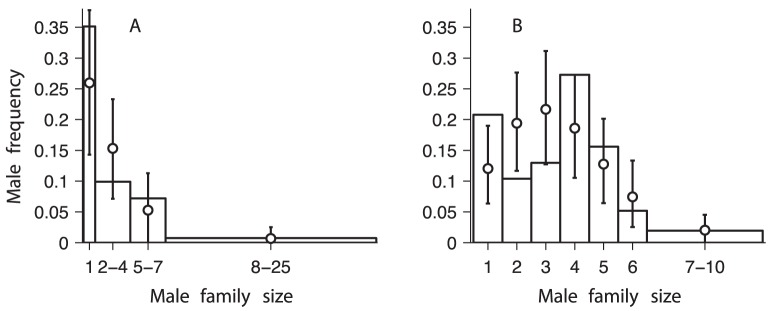
Distribution of number of progeny attributed to each sire. Bars in panel **A** show the empirical data obtained under experimental conditions; the data correspond to six broods, two of which have size 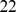, and the remaining four have sizes 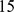, 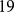, 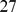, and 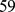. Bars in panel **B** show the results from females mated in the field, data taken from [10]; the data correspond to four broods of sizes 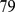, 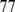, 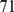, and 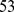. The width of the bins are chosen so that the expected number of counts in each bin is not smaller than 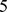. The probability assigned to each bar is proportional to the bar area. Symbols and error bars show the result of the best fit to the experimental data, together with their 95% confidence intervals: 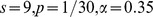 in (**A**), and 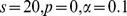 in (**B**). We simulated 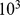 independent realisations of the mating process.

In Fig. 3 **A** we show the histogram of male family sizes obtained experimentally and in Fig. 3 **B** we show similar data collected from wild females in natural habitats. For both sets of data we use computer simulations in order to find the parameters in our model resulting in male family sizes that are closest to those empirically observed. In the computer simulations we vary the number of available mates 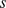, the probability 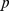, and the level of dominance of a dominant male, 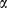. For the females mated in the lab, we vary the parameter 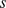 from 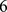 to 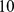, and for the wild females, we vary 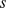 from 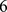 to 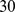. In both cases, we vary the parameter 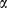 from 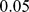 to 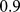 in steps of 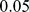, and the inverse of parameter 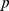 from 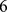 to 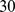 in steps of 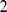, and we test 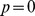 as well. For each set of test values of 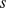, 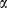, and 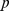, we simulate mating of six females (four for wild snails) and we generate broods of sizes corresponding to that in the empirical data. By simulating the process of mating 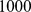 times for each set of test parameters, we compute the average probability of obtaining a given male family size. Using these data, we apply a chi-square test to quantify the difference between the empirical and simulated data for each set of test parameters. The best fits obtained are shown in Fig. 3 **A**–**B** by circles, and they correspond to 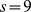, 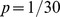, 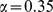 (Fig. 3 **A**), and to 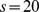, 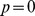, 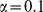 (Fig. 3 **B**). We discuss these fits in Section **Discussion and conclusions**. The results shown in Fig. 3 suggest that our mating model describes empirical data well, but the agreement is not perfect. Possible improvements of our model are discussed in Section **Discussion and conclusions**.

To address the question of how multiple paternity affects genetic variation and structure in our subdivided population, we analyse genetic diversity under the colonisation model described above. We analyse separately two phases of population dynamics on each island: initial colonisation, and the steady state that develops once the colonisation phase is over. For a given island, we compute the expected heterozygosity in the generation when the island is colonised (colonisation phase), as well as the expected heterozygosity in the steady state. The corresponding analytical computations are described in detail in **Appendix S2** and **Appendix S3**. We also study temporal changes of heterozygosity under our model by computer simulations. In the following two subsections we present separately the results for the colonisation phase and for the steady state.

### Colonisation phase

We compute the heterozygosity in the colonisation phase analytically using a coalescent approach [26]. Recall that at a given island, the colonisation-phase heterozygosity corresponds to the island heterozygosity in the generation when the island is colonised. Note that the population-size history of the population on island 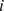 at the time when this island is colonised can be represented as a sequence of 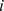 bottlenecks. In our model, each of the bottlenecks lasted one generation, and the most recent bottleneck occurred when the first colonisers (founder females) came to island 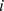. Note that there are 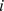 bottlenecks, because 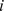 is the number of colonisation events that the population ancestral to that on island 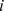 went through before the island was colonised. We derive an analytical result upon assuming that the migration rate is small, 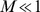. Under this assumption, colonisation of empty islands occurs rarely, but when it does, an island typically receives a single founder female. The resulting heterozygosity in island 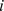 in the colonisation phase, 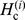, is (see **Appendix S2**):
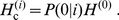
(5)Here, 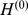 is the mainland heterozygosity, and 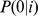 is the probability that the most recent common ancestor of two alleles sampled from the newly established population in island 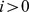 stems from an allele that was born on the mainland (see **Appendix S2**):
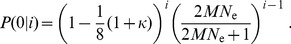
(6)It follows from Eqs. (5) and (6) that the dependence of the heterozygosity in the colonisation phase on the degree of multiple paternity 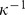 can not be expressed only in terms of the effective population size. This suggests that the degree of multiple paternity has possibly a larger effect on the colonisation-phase heterozygosity in subpopulations, than on the heterozygosity of freely mixing populations.

**Figure 4:**
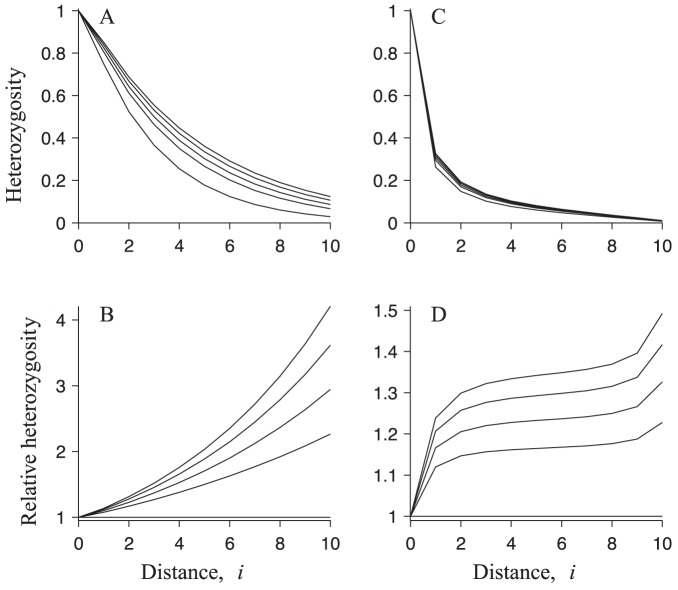
Analytically computed heterozygosity during colonisation and in the steady state. (**A**) Heterozygosity during colonisation as a function of distance from the mainland. The lines shown from top to bottom correspond to: 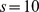, 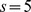, 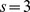, 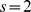, and 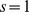. (**B**) Heterozygosity during colonisation relative to its corresponding value for 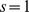 as a function of distance from the mainland. The order of lines corresponds to that in **A**. (**C**) and (**D**) Same as in (**A**) and (**B**), respectively, but for the steady state. Remaining parameters: all available males are on average equally successful, 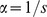, the mainland heterozygosity is set to unity, the scaled female migration rate is 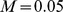, the number of females in each populated island is 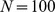, the probability that two eggs are fertilised by the same sperm package is 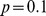, and the number of islands is 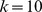.

In Fig. 4 **A** we show how the colonisation-phase heterozygosity depends on distance from the mainland. We observe two expected features. First, the colonisation-phase heterozygosity decays as distance from the mainland increases. Second, at any distance from the mainland, multiple paternity results in higher heterozygosity than single paternity. We find that mating two males (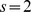) increases the values of single-paternity heterozygosity by 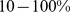 for the parameters used in Fig. 4 **A**, and mating ten males (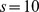) increases the values of single-paternity heterozygosity by 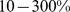, depending on distance from the mainland (Fig. 4 **B**). The largest increase is observed at the island furthest from the mainland. It is interesting to note that mating more than about 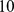 males only marginally increases the heterozygosity (results not shown), as found in the case of freely mixing populations (see our description of the mating model).

We note that the results of our computer simulations (see Fig. S1 **A**–**C**) agree well with the analytical results for low migration rates. For large migration rates (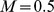, i. e. 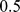 females on average per generation) by contrast, the simulations assume somewhat higher values than the theory. The reason for this deviation is that for 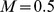 it is probable that more than one founder female come to an empty island to establish the population, and, consequently, will contribute with more genetic variation than just one founder female. For natural populations of *L. saxatilis* it has been estimated that 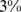 of empty islands receive a migrant each year [12]. This estimate is close to the lower value of 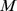 used in our simulations (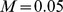).

### Steady state

We show in **Appendix S3** how to compute the heterozygosity in the steady state at distance 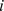 from the mainland using recursion relations (this is a standard procedure, see for example [19], [21]). Note that this derivation does not require 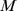 to be small. In Fig. 4 **C**, we show how the resulting steady-state heterozygosity depends on distance from the mainland. As in the colonisation phase, the steady-state heterozygosity decreases as distance from the mainland increases. Also, by increasing the degree of multiple paternity, the heterozygosity at a given island increases (this effect saturates at 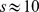, results not shown). In contrast to the strong effect of multiple paternity during colonisation, the effect is substantially smaller in the steady state. We find that the single-paternity heterozygosity in the steady state increases by 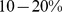 for 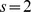, and by 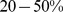 for 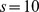 (Fig. 4 **D**). As in the colonisation phase, the largest increase is observed at the island furthest from the mainland.

**Figure 5:**
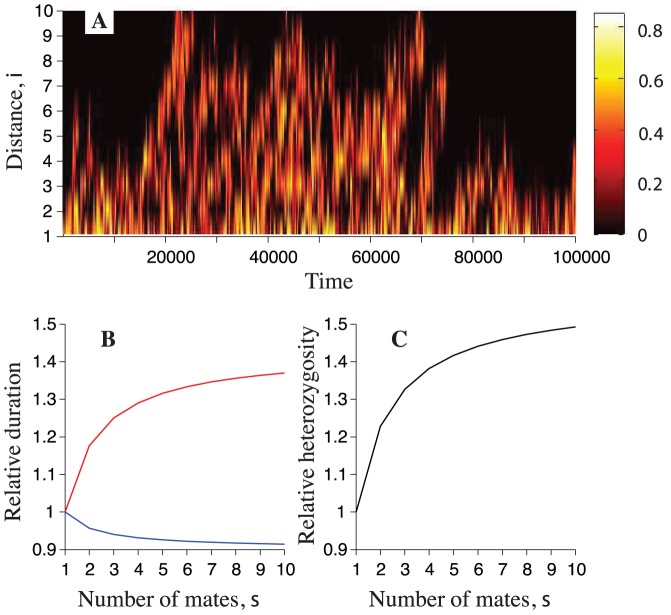
Temporal fluctuations of heterozygosity. (**A**) Heterozygosity as a function of distance from the mainland and of time (single realisation of the model described). Mainland is not shown. The data correspond to 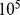 generations after the initial 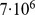 generations. The number of available mates is 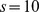. (**B**) Analytically computed durations of low-and high-heterozygosity phases (blue, and red) relative to their corresponding values for 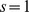. (**C**) Analytically computed steady-state heterozygosity (black) relative to its corresponding value for 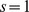. Remaining parameters are as in Fig. 4 **C**.

In addition, we examined the variation in heterozygosity over consecutive generations in a particular realisation of our model. We find that the heterozygosity shows strong temporal fluctuations (Fig. 5 **A**). Notably, the fluctuations are strongest furthest from the mainland, with periods of high diversity separated by long periods of near or complete fixation. Hence the distribution of heterozygosity at large distances from the mainland is bimodal. The heterozygosity is expected to have a bimodal distribution if genetic variation is introduced at a very small rate (see, for example, [27] and references cited therein).

In what follows, we analyse how the durations of the phases of low and high heterozygosity are affected by multiple paternity. We consider values of the heterozygosity smaller than 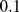 to be low, and values of the heterozygosity larger than 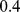 to be high. The minimum value for the high phase (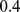) is chosen since the typical maximum value that the heterozygosity has at the island furthest from the mainland is 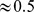 for the parameters chosen in Fig. 5 **A**. Note that the maximum value of the heterozygosity at a locus with only 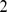 allelic types is equal to 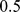.

Using the results of our computer simulations, we compute the average durations of low- and high-heterozygosity phases at the island furthest from the mainland. We also derive corresponding analytical results under the assumption that the scaled migration rate 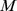 is small (see **Appendix S4**). For island 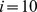 we show in Fig. 5 **B** (see also Fig. S2 **B**, **D**, **F**) the durations of low- and high-heterozygosity phases relative to their corresponding values for a single mate (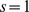). Fig. 5 **B** shows that multiple paternity prolongs the duration of the high-heterozygosity phase, and decreases the duration of the low-heterozygosity phase. For example, the high-heterozygosity phase for the highest level of multiple paternity shown (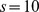) is prolonged by around 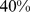 compared to its value under single paternity (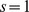). The low-heterozygosity phase is shortened by only around 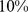 for 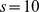 (Fig. 5 **B**). For comparison, Fig. 5 **C** shows the steady-state heterozygosity relative to its corresponding value for a single mate (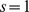). In conclusion, multiple paternity promotes heterozygosity by prolonging the duration of peaks of variation that reach the most distant islands.

## Discussion and Conclusions

In this study we analysed the effect of multiple paternity on genetic diversity over spatial and temporal scales in a population living in islands distributed in one dimension. We quantified the effect of multiple paternity during the colonisation of the semi-isolated islands and in the steady state developed after the colonisation phase. Our conclusions given below can be generalised to a population inhabiting relatively small patches that are partly isolated from each other and approximately linearly distributed, such as, rocky outcrops along a sandy coast, sandy beaches along a rocky coast, distinct patchy habitats along a stream or a river, patchy habitats found at a specific mountain altitude, small lakes at an increasing distance from a larger lake, etc..

We introduced a mating model which allows for different levels of multiple paternity in a population. As expected, we found that by increasing the degree of multiple paternity within our mating model, the effective population size increases, and thus the heterozygosity increases. Yet, the consequences of multiple paternity on heterozygosity in relatively small and semi-isolated populations are substantial, which is summarised in the following.

At a given distance from the mainland, populations with high degree of multiple paternity establish higher heterozygosity than populations with low degree of multiple paternity. In the steady state, this effect is expected, since the heterozygosity in the steady state depends on the effective population size and the rate of income of new genetic material to a given habitat. It is also expected that this effect saturates at the same degree of multiple paternity as the effective population size of a freely mixing population, which is consistent with our findings. By comparing the effect of multiple paternity on the heterozygosity in the steady state to that on the heterozygosity in the colonisation phase, we find that the latter is substantial. This difference between the steady state and the colonisation phase is explained in the following. Upon the arrival of founder females to an empty island, the carrying capacity of an island is reached within a single generation. Therefore, such a newly established population receives genetic material of most males that the founder females were inseminated by. By contrast, in the steady state, all mothers present in an island contribute to the population in the next generation, and hence the impact of immigrant females to the next generation is rather small. From this reasoning, we find that it is possible that the effect of multiple paternity upon heterozygosity during colonisation might decrease if the growth rate of the island populations up to the carrying capacity were less than infinite (as assumed in our model).

The heterozygosity at distances far from the mainland fluctuates significantly. Long periods of almost complete loss of genetic variation are interrupted by bursts of high heterozygosity, and we quantified how this effect is boosted by multiple paternity. The durations of high- and low-heterozygosity phases could be an important survival factor in natural populations. For example, the low-heterozygosity phase could be disadvantageous if a malignant disease appears in the population, assuming that only a particular mutation (not present in the population, or being rare) can survive the disease. Moreover, in some species evidence was found for selection acting against homozygous individuals [28]. The wave-like nature of the spread of new alleles from the mainland population is also seen in the correlation of genetic diversity at neighbouring islands. We find that the correlation between heterozygosities at a pair of nearest-neighbouring islands increases as distance from the mainland increases (results not shown). These results suggest intermittent bursts of genetic diversity in remote islands, an effect which becomes stronger as the degree of multiple paternity increases. We note that a similar trend is expected in our model even when the process of mutation on islands is explicitly included in simulations, as long as the migration rate is not too small. This is because for a given value of the scaled mutation rate on the mainland, the scaled mutation rate on islands inevitably approaches zero (the mainland is infinitely larger than islands).

The conclusions given above are confirmed by our computer simulations. In order to minimise computing time during simulations, we assumed that the mainland heterozygosity is equal to unity, which guarantees that whenever a migrant from the mainland comes to the first island, it carries genetic material that previously did not exist on any of islands (and thus the population dynamics on the mainland does not need to be simulated explicitly). However, we emphasise that the conclusions given above qualitatively do not depend on the value of heterozygosity on the mainland.

We note that, unlike in our model, it is possible that the rate of successful colonisation in natural habitats is smaller than the rate of migration. For example, if an immigrant female carries a small number of progeny, her progeny alone may not be enough to colonise an empty island successfully. By allowing for the rate of successful colonisation to be smaller than the rate of migration in the colonisation model, the steady-state values of heterozygosity remain equal to those obtained under the assumption that the colonisation and migration rates are the same (as in our model). The values of heterozygosity during colonisation, by contrast, are expected to differ from those found here.

In order to determine how realistic our mating model is, we compared the male family sizes of female broods obtained within our model, to empirically observed family sizes in populations of *L. saxatilis* from natural habitats and under experimental conditions. By computing chi-square values, we found that empirical male family size distributions were in agreement with our mating model, that is our mating model could not be rejected. The best-fitting parameters for the data obtained under experimental conditions indicated that the number of available mates for a female is on average less than the number of males that a female was surrounded by in her aquarium. This might be due to the success of mates is variable, so that some of the available mates did not mate at all during the time of the experiments (males were removed after eight weeks). Moreover, many males are likely to have mated multiple times during the experiment, while some may have mated just a few times, and for those males it is possible that they did not sire any offspring at all. In addition, females may exhibit cryptic choice of sperm. We also found that the best-fitting parameters for the data obtained under experimental conditions indicated fewer matings than brood sizes, suggesting that some of the eggs were fertilised by sperm retained between matings. By contrast, the corresponding empirical distribution in natural populations was best fitted by assuming an unlimited number of matings (i. e. no sperm retention). This is consistent with the high density of snails observed in the wild. However, both empirical distributions showed an excess of males with a single progeny compared to the mating model with the best-fitting parameters. This discrepancy could be because the success of available mates in natural conditions is highly variable, or a female exhibits cryptic choice of sperm.

We note that for the females mated in the lab, only the offspring that could uniquely be assigned to a given sire were used in the analysis. This resulted in removing 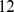 out of 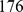 offspring from the parentage assignment. While this could in principle bias offspring towards males with more unique alleles, we estimated that the likelihood to inherit the same set of alleles from two males is very small for all pairs of males (the majority of pairs of males have at least two loci with no shared alleles). This also means that it is unlikely that genotyping errors would lead to misassignments. By contrast, for the data corresponding to wild females, genotypes of possible fathers were unknown. They were estimated using a maximum likelihood approach [10]. Since it is possible that there were a number of fathers with similar genotypes, the maximum likelihood method in [10] could have underestimated the true number of fathers. In particular, some of the large male families found might have consisted of a number of smaller families, as a given large male family could have belonged to multiple alike fathers. As a consequence, we expect that the assignment in this case was biased so that not only the total number of fathers was underestimated, but also the number of small male families. However, it was argued in [10] that, since the estimated number of fathers of the four broods analysed was large, it is likely that these estimates are very close to the corresponding true number of fathers. For this reason, we expect that the effect of genotyping errors in this case would also be small.

Since mating is considered to be costly [10], we raise the question whether or not mating multiple males is an evolutionary strategy of *L. saxatilis* to increase the heterozygosity. Recall that we estimated that in natural populations of *L. saxatilis* the probability that two eggs are fertilised by the same sperm package is likely to be very small. Under our model, this probability is equal to zero if each sperm package fertilises one egg, or if the actual number of sperm packages a female receives during her reproductive cycle is very large. If the latter applies, we find that it is unlikely that the heterozygosity increase is the main reason for the extreme multiple paternity in this species. As earlier suggested, it seems likely that the cost of rejecting an intercourse is higher than the cost of accepting it, and a consequence of convenience polyandry [10].

In summary, this study can be used to quantify the gene flow between partly isolated natural populations using allelic frequencies at a number of neutral loci. In particular, since the spatial patterns of heterozygosity during the colonisation phase differ from those in the steady state, this study can be used for determining whether or not the colonisation process started recently.

Furthermore, since our results show that the heterozygosity exhibits extreme fluctuations in populations founded through repeated founder events, we raise the question of whether similar fluctuations can be observed at any given time at neutral loci sampled genome-wide. In order to answer this and related questions, the effect of recombination needs to be analysed. Since island populations in our model experience at least one severe bottleneck, we expect that the degree of linkage disequilibrium in the colonisation phase is constant over a range of genetic distances, as shown in [29]. However, how multiple paternity affects linkage disequilibrium during colonisation and in the steady state is yet to be investigated. It would also be interesting to analyse how selection combined with recombination affects genetic diversity in a subdivided population. Such results would provide an advance in the endeavor of identifying genes under selection, and especially, the genes underlying speciation [30]–[32].

All simulations in this study were written in Matlab. The simulations as well as their raw results used in this study are freely available upon request.

## Supporting Information

Appendix S1 (PDF)

Appendix S2 (PDF)

Appendix S3 (PDF)

Appendix S4 (PDF)

Figure S1
**Heterozygosity in the colonisation phase, and in the steady state.** (**A**)–(**C**) Colonisation phase. The analytical results are shown as solid lines, and the results of computer simulations are shown as symbols. The number of available mates is 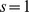 (blue), 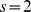 (red), 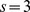 (green), 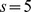 (magenta), and 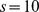 (black). Averages are over 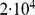 realizations of the process of colonisation of empty islands. (**D**)–(**F**) Same as in (**A**)–(**C**) but for the steady state. In panel **D**, averaging is done over 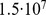 generations (the initial 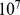 generations being discarded), and over three independent realisations of our model. In panel **E**, averages are over 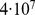 generations (the initial 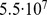 generations being discarded) and over four independent realisations of our model. In panel **F**, averaging is done over 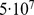 generations (the initial 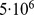 generations being discarded) and over five independent realisations of our model. Remaining parameters used: all available males are on average equally successful, 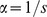, mainland heterozygosity 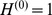, number of islands 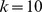.(EPS)

Figure S2
**Low- and high-heterozygosity phases.** (**A**) Illustration of the method used to determine the duration of low- and high-heterozygosity phases. The panel shows the heterozygosity of the population on island 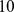 as a function of time. The heterozygosity represented in terms of the low and high phases is shown by the magenta line. The black line depicts the result of computer simulation. The data shown correspond to those in Fig. 5 **A** in the main text. (**B**), (**D**), and (**F**) Durations of low- and high heterozygosity phases relative to their corresponding values for 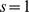 (blue and red, respectively) in island 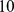. (**C**), (**E**), and (**G**) Steady-state heterozygosity in island 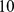 relative to its corresponding value for 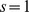. The results of computer simulations are shown as symbols, and the analytical results are shown as solid lines. The parameters in **B**, and **C** correspond to those in Fig. S1 **D**. The parameters in **D**, and **E** correspond to those in Fig. S1 **E**. The parameters in **F**, and **G** correspond to those in Fig. S1 **F**.(EPS)
