# Supplementary material for: The Effect of Multiple Paternity on Genetic Diversity of Small Populations during and after Colonisation
Source: PLoS One. 2013 Oct 28;8(10):e75587. doi: 10.1371/journal.pone.0075587 (PMC3810386; doi:10.1371/journal.pone.0075587)
Supplement: Appendix S1 — (PDF) [file pone.0075587.s001.pdf]

Appendix S1

In this appendix we compute the effective population size under the mating model introduced in the main text. We assume that the population is diploid, isolated, well-mixed and that it consists of $N_{\rm f}$ females, and $N_{\rm m}$ males. We assume $N_{\rm f} \gg 1$ and $N_{\rm m} \gg 1$. In a diploid population with effective population size $N_{\rm e} \gg 1$, the population homozygosity F_τ in generation τ is given by:

$$F_\tau \approx \frac{1}{2N_{\rm e}}\epsilon_\tau + (1 - \frac{1}{2N_{\rm e}})\chi_\tau \ . \tag{S1}$$

Here ϵ_τ is the inbreeding coefficient which stands for the probability that two alleles within a single randomly chosen individual are identical at time τ. The second term, χ_τ, is the coancestry, that is the probability that two alleles sampled in generation τ from two different randomly chosen individuals are identical. In what follows, we compute F_τ under our mating model, and thereafter we use Eq. (S1) to determine the corresponding effective population size. We assume that mutation rate per generation per allele is $\mu \ll 1$, and we employ the infinite-alleles model [1]. Our calculations are based on the approach used in [2].

Under the assumption that all females are on average equally successful in producing offspring, it follows that the probability for two offspring to come from the same female, $P_{\rm f}$, is equal to $P_{\rm f} = N_{\rm f}^{-1}$. Let κ be the probability that two offspring coming from the same mother also share a father. The probability that two offspring come from the same male, $P_{\rm m}$, is thus:

$$P_{\rm m} = P_{\rm f}\kappa + (1 - P_{\rm f})\frac{1}{N_{\rm m}} \ , \tag{S2}$$

where $N_{\rm m}^{-1}$ is the probability that two offspring having different mothers stem from the same father. The probability κ has two contributions: the probability that two offspring come from the same sperm package, p, and the probability that two offspring do not come from the same sperm package but they come from the packages coming from the same male, $(1-p)(\alpha^2 + (1-\alpha)^2/(s-1))$. It follows that κ is given by

$$\kappa = p + (1-p)\left(\alpha^2 + \frac{(1-\alpha)^2}{s-1}\right) \ , \tag{S3}$$

where p depends on the number of active sperm packages received by each female, l, and on the number of eggs fertilised by a single sperm package during its persistence time, $N_{\rm eggs}$

$$p = \frac{l\binom{N_{\rm eggs}}{2}}{\binom{lN_{\rm eggs}}{2}} \ . \tag{S4}$$

If all sperm packages persist until the end of the reproductive cycle, all eggs are fertilised after all sperm packages have been collected, and sperm packages are chosen with replacement to fertilise eggs, it follows that

$$p = \frac{1}{l} \ . \tag{S5}$$

As mentioned in the main text (see Section **Mating model**), we take κ^{-1} as a measure of the degree of multiple paternity. The case of single paternity corresponds to $\kappa = 1$.

Using the expressions for P_m and P_f, we compute ϵ_τ, and χ_τ recursively. Under our model we find

$$\epsilon_\tau = (1-\mu)^2 \chi_{\tau-1} \ , \tag{S6}$$

$$\chi_\tau = (1-\mu)^2 \Bigg\{ \frac{1}{4N_\mathrm{f}} \left[\frac{1+\epsilon_{\tau-1}}{2}(1+\kappa) + (3-\kappa)\,\chi_{\tau-1} \right] + \frac{1}{4}\left(1-\frac{1}{N_\mathrm{f}}\right) \left[\frac{1+\epsilon_{\tau-1}}{2N_\mathrm{m}} + \left(4-\frac{1}{N_\mathrm{m}}\right)\chi_{\tau-1} \right] \Bigg\} \ . \tag{S7}$$

After rearranging the terms in Eqs. (S6)-(S7), and keeping only the leading-order terms, we obtain:

$$\epsilon_\tau = (1-2\mu)\chi_{\tau-1} \ , \tag{S8}$$

$$\chi_\tau = \frac{1}{8}\left(\frac{1+\kappa}{N_\mathrm{f}} + \frac{1}{N_\mathrm{m}}\right) + \frac{1}{8}\left(\frac{1+\kappa}{N_\mathrm{f}} + \frac{1}{N_\mathrm{m}}\right)\epsilon_{\tau-1} + \left[1 - \frac{2}{8}\left(\frac{1+\kappa}{N_\mathrm{f}} + \frac{1}{N_\mathrm{m}}\right)\right]\chi_{\tau-1} - 2\mu\chi_{\tau-1} \ . \tag{S9}$$

Using Eqs. (S8)-(S9) we find the standard expression for the steady-state homozygosity

$$F = \frac{1}{1+\theta} \ . \tag{S10}$$

Here, as usual, $\theta = 4\mu N_\mathrm{e}$ is the scaled mutation rate. The effective population size N_e is given by

$$N_\mathrm{e} = 4\left(\frac{1+\kappa}{N_\mathrm{f}} + \frac{1}{N_\mathrm{m}}\right)^{-1} \ . \tag{S11}$$

In the case $N_\mathrm{m} = N_\mathrm{f} = N$ (as assumed for island populations in our colonisation model), Eq. (S11) becomes:

$$N_\mathrm{e} = \frac{4N}{2+\kappa} \ . \tag{S12}$$

Upon setting $\kappa = 0$, Eq. (S12) reduces to $N_\mathrm{e} = 2N$, that is the effective population size becomes equal to the census population size. This is the maximum value that N_e can acquire in a freely mixing population of census size $N_\mathrm{m} + N_\mathrm{f}$.

References

1. Kimura M, Crow JF (1964) The number of alleles that can be maintained in a finite population. Genetics 49: 725-738.

2. Balloux F, Lehmann L (2003) Random mating with a finite number of matings. Genetics 165: 2313-2315.
