# Supplementary material for: The Effect of Multiple Paternity on Genetic Diversity of Small Populations during and after Colonisation
Source: PLoS One. 2013 Oct 28;8(10):e75587. doi: 10.1371/journal.pone.0075587 (PMC3810386; doi:10.1371/journal.pone.0075587)
Supplement: Appendix S2 — (PDF) [file pone.0075587.s002.pdf]

Appendix S2

In this appendix we compute the population heterozygosity in the colonisation phase. As mentioned in the main text (see Section **Stepping-stone colonisation model**), it is assumed that only the mainland is populated initially, and that its population size is constant in time. Moreover, we assume that the mainland population exists for a long time before colonisation begins (this occurs at generation $\tau = 0$). We denote the colonisation-phase heterozygosity on the mainland by $H^{(0)}$.

In order to find an expression for the heterozygosity in island i in the generation when island i is colonised, $H_{\rm c}^{(i)}$, we use the coalescent approach. Recall that a populated island is assumed to consist of N males and N females (N is large), and it is assumed that this population size is much smaller than that of the mainland. Moreover, in the following we assume that the migration rate M is small, $M \ll 1$. The average time between two successive founder events is thus long, and typically one founder female arrives at an empty island (the probability that two females come simultaneously is of order M^2, negligible for $M \ll 1$). Under this assumption, the ancestral population size of the newly established population in island i can be represented by a sequence of i bottlenecks, such that each bottleneck lasts for one generation (since the founder female gives rise to $2N$ offspring), and the time between two successive bottlenecks is on average M^{-1} generations long. Upon expressing the generation index τ by t such that $\tau = \lfloor 2tN_{\rm e} \rfloor$, where $N_{\rm e}$ is the effective population size given by Eq. (S12) in **Appendix S1**, the waiting time between two successive founder events is approximately exponentially distributed with mean

$$(2MN_{\rm e})^{-1} \ . \tag{S13}$$

In order to compute the heterozygosity, we note that in our model the mainland acts as the only source of genetic variation. This allows us to argue the following. First, if the most recent common ancestor (MRCA) of two alleles sampled randomly from the newly established population in island i was born on island $j < i$ ($j \neq 0$), the two alleles sampled are identical. Second, if the MRCA was born on the mainland, the two alleles are expected to be identical with probability $F^{(0)} = 1 - H^{(0)}$. Therefore, in order to compute $H_{\rm c}^{(i)}$, it suffices to determine the probability that the MRCA of two lines sampled from the newly established population in island i stems from an allele that was born on the mainland, $P(0|i)$.

The probability $P(0|i)$ has two contributions. The first contribution is the probability that the MRCA of two alleles sampled in island i is not found during a bottleneck. We find this to be equal to $1 - \frac{1}{8}(1 + \kappa)$. The second contribution is the probability that the MRCA of two alleles is not found between two successive bottlenecks. This term is equal to $2MN_{\rm e}(2MN_{\rm e} + 1)^{-1}$. It follows that $P(0|i)$ is given by

$$P(0|i) = \left(1 - \frac{1}{8}(1+\kappa)\right)^{i} \left(\frac{2MN_{\rm e}}{2MN_{\rm e}+1}\right)^{i-1} \ . \tag{S14}$$

Therefore, the colonisation-phase heterozygosity in island i is:

$$H_{\rm c}^{(i)} = P(0|i) H^{(0)} \ . \tag{S15}$$

Note that for the case described here, the population size switches between $2N$ (N males and N females during the waiting time before the colonisation of the next island) and unity (one inseminated mother during a bottleneck). Therefore, $P(0|i)$ depends on κ not only through $N_{\rm e} = 4N(2 + \kappa)^{-1}$, but also through the first factor in Eq. (S14).

The heterozygosity in the colonisation phase for different parameters of our model is shown in Fig. S1**A**-**C**. The solid lines in Fig. S1**B** correspond to the solid lines in Fig. 4**A** in the main text. We see that the agreement between Eq. (S15) and the results of computer simulations is good for $M = 0.05$, whereas for $M = 0.5$, Eq. (S15) underestimates the results of computer simulations. This is discussed in the main text (see Section **Results**).
