# Supplementary material for: The Effect of Multiple Paternity on Genetic Diversity of Small Populations during and after Colonisation
Source: PLoS One. 2013 Oct 28;8(10):e75587. doi: 10.1371/journal.pone.0075587 (PMC3810386; doi:10.1371/journal.pone.0075587)
Supplement: Appendix S3 — (PDF) [file pone.0075587.s003.pdf]

Appendix S3

In this appendix, the steady state within the model introduced in the main text is analysed. Expressions for the steady-state heterozygosity on islands at distance $i = 1, \dots, k$ from the mainland are derived under the assumption that all islands are populated (that is, the colonisation phase is over). As discussed in **Appendix S1**, the inbreeding coefficient in generation τ in island i, $\epsilon_\tau^{(i)}$, and the coancestry, $\chi_\tau^{(i)}$, contribute to the homozygosity $F_\tau^{(i)}$ in island i in generation τ. The coancestry between islands i, and j is equal to the inter-island homozygosity, and for this case we use the notation $\chi_\tau^{(i,j)} \equiv F_\tau^{(i,j)}$ ($i \neq j$).

As mentioned in **Appendix S2**, we assume that the mainland is the only source of genetic variation. All habitats are assumed to have equal numbers of males and females. The population size on the islands $i = 1, \dots, k$ is assumed to be large (and equal to $2N$), but much smaller than that of the mainland. As before, the heterozygosity on the mainland in generation $\tau = 0$ is denoted by $H^{(0)}$.

According to the spatial model introduced in the main text, the female-migration rate per island per generation is $2M$ for islands $i = 1, \dots, k-1$, whereas for the mainland and for the island furthest from the mainland it is equal to M. Since the population size on the mainland is much larger than that of a populated island, the process of migration does not affect genetic variation on the mainland. Therefore, we have $H_\tau^{(0)} = H^{(0)}$. For the island populations, we consider separately the mainland-island, the inter-island and the intra-island homozygosity. First, we treat sampling from two distinct populations, $i \neq j$. Second, we consider the case of sampling within a single island $i = 1, \dots, k$. Our calculations given below are based on the approach employed in [1].

The mainland-island homozygosity $F_{\tau+1}^{(i,j)}$ for $i = 0$, $0 < j \leq k$ satisfies the following recursion:

$$F_{\tau+1}^{(0,j)} = (1 - m + \delta_{j,k}\frac{m}{2})F_\tau^{(0,j)} + \frac{m}{2}\left(F_\tau^{(0,j-1)} + (1-\delta_{j,k})F_\tau^{(0,j+1)}\right) \ . \tag{S16}$$

Here $m = 2M/N \ll 1$ is the migration rate per island per female per generation, and $\delta_{j,k}$ is equal to unity when $j = k$, and it is zero otherwise. The inter-island homozygosity for $0 < i < k$, $0 < j < k$, $i \neq j$, obeys:

$$\begin{aligned} F_{\tau+1}^{(i,j)} = (1-m)\Bigg[&(1-m)F_\tau^{(i,j)} + m\chi_\tau^{(i)}\left(\delta_{i,j-1} + \delta_{i,j+1}\right) \\ &+ \frac{m}{2}(1-\delta_{i,j-1})\left(F_\tau^{(i,j-1)} + F_\tau^{(i+1,j)}\right) \\ &+ \frac{m}{2}(1-\delta_{i,j+1})\left(F_\tau^{(i,j+1)} + F_\tau^{(i-1,j)}\right)\Bigg] + O(m^2) \ . \end{aligned} \tag{S17}$$

Lastly, when $i = k$, $0 < j < k$, we find

$$\begin{aligned} F_{\tau+1}^{(k,j)} &= \big(1-\frac{m}{2}\big)(1-m)\,F_{\tau}^{(k,j)} \\ &+ \frac{m}{2}(1-\frac{m}{2})\Big(F_{\tau}^{(k,j-1)}(1-\delta_{k,j-1})+\chi_{\tau}^{(k,j-1)}\delta_{k,j-1}\Big) \\ &+ \frac{m}{2}(1-\frac{m}{2})\Big(F_{\tau}^{(k,j+1)}(1-\delta_{k,j+1})+\delta_{k,j+1}\chi_{\tau}^{(k,j+1)}\Big) \\ &+ \frac{m}{2}(1-m)\Big(F_{\tau}^{(k-1,j)}(1-\delta_{k,j+1})+\chi_{\tau}^{(k-1,j)}\delta_{k,j+1}\Big)+O(m^2)\,. \end{aligned} \tag{S18}$$

The inbreeding coefficient of the population on island $0 < i < k$ satisfies:

$$\epsilon_{\tau+1}^{(i)} = (1-m)\,\chi_{\tau}^{(i)} + \frac{m}{2}\chi_{\tau}^{(i-1)} + \frac{m}{2}\chi_{\tau}^{(i+1)}\ , \tag{S19}$$

and the coancestry is given by:

$$\begin{aligned} \chi_{\tau+1}^{(i)} = (1-m)^2\frac{1}{N(1-m)}&\left(\frac{1+\epsilon_{\tau}^{(i)}}{8}(1+\kappa)+\frac{1-\kappa}{4}\chi_{\tau}^{(i)}+\frac{\chi_{\tau}^{(i)}}{2}\right) \\ +(1-m)^2(1-\frac{1}{N(1-m)})&\left(\frac{3}{4}\chi_{\tau}^{(i)}+\frac{1+\epsilon_{\tau}^{(i)}}{8N}+(1-\frac{1}{N})\frac{\chi_{\tau}^{(i)}}{4}\right) \\ +m(1-m)&\left(F_{\tau}^{(i,i+1)}+F_{\tau}^{(i,i-1)}\right)+O(m^2)\ . \end{aligned} \tag{S20}$$

For the island furthest from the mainland ($i = k$), we have:

$$\epsilon_{\tau+1}^{(k)} = \left(1-\frac{m}{2}\right)\chi_{\tau}^{(k)} + \frac{m}{2}\chi_{\tau}^{(k-1)}\ , \tag{S21}$$

$$\begin{aligned} \chi_{\tau+1}^{(k)} = (1-\frac{m}{2})^2\frac{1}{N(1-\frac{m}{2})}&\left(\frac{1+\epsilon_{\tau}^{(k)}}{8}(1+\kappa)+\frac{1-\kappa}{4}\chi_{\tau}^{(k)}+\frac{\chi_{\tau}^{(k)}}{2}\right) \\ +(1-\frac{m}{2})^2(1-\frac{1}{N(1-\frac{m}{2})})&\left(\frac{3}{4}\chi_{\tau}^{(k)}+\frac{1+\epsilon_{\tau}^{(k)}}{8N}+(1-\frac{1}{N})\frac{\chi_{\tau}^{(k)}}{4}\right) \\ +m(1-\frac{m}{2})&F_{\tau}^{(i,i-1)}+O(m^2)\ . \end{aligned} \tag{S22}$$

In what follows we keep only the leading order terms in Eqs. (S16)-(S22). Moreover, we use a scaled time t, where $\tau = \lfloor 2N_{\rm e}t \rfloor$, and $N_{\rm e}$ is the effective population size of an island population under our mating model (see Eq. (S12) in **Appendix S1**). In these units of time, we denote the homozygosity at time t in island i by $F^{(i)}(t)$.

We conclude this appendix by giving differential equations (with only the leading order terms) for the mainland-island homozygosity, then for the inter-island homozygosity and, lastly, for the intra-island homozygosity. For the mainland-island homozygosity we find:

$$0 = -\,\partial_t F^{(0,i)}(t) + M_{\rm e}\left(F^{(0,i+1)}(t) + F^{(0,i-1)}(t) - 2F^{(0,i)}(t)\right)\ . \tag{S23}$$

Here, $M_\mathrm{e} = 2MN_\mathrm{e}/N$, and it is assumed that $i < k$. For $i = k$, we have:

$$0 = -\partial_t F^{(0,k)}(t) + M_\mathrm{e}\left(F^{(0,k-1)}(t) - F^{(0,k)}(t)\right) \ . \tag{S24}$$

For the inter-island homozygosity between islands i and j, where $0 < i < k$, $0 < j < k$, $j \neq i$, we find:

$$\begin{aligned} 0 = &- \partial_t F^{(i,j)}(t) + M_\mathrm{e}\left(1 - \delta_{i-1,j}\right)\left(F^{(i,j+1)}(t) + F^{(i-1,j)}(t)\right) \\ &+ M_\mathrm{e}\delta_{i-1,j}\left(F^{(i)}(t) + F^{(i-1)}(t)\right) + M_\mathrm{e}\left(1 - \delta_{i+1,j}\right)\left(F^{(i,j-1)}(t) + F^{(i+1,j)}(t)\right) \\ &+ M_\mathrm{e}\delta_{i+1,j}\left(F^{(i)}(t) + F^{(i+1)}(t)\right) - 4M_\mathrm{e}F^{(i,j)}(t) \ . \end{aligned} \tag{S25}$$

For $i = k$, $0 < j < k$, we obtain:

$$0 = -\partial_t F^{(k,j)}(t) + M_\mathrm{e}\left(F^{(k-1,j)}(t) - 2F^{(k,j)}(t)\right) \ . \tag{S26}$$

Finally, for the homozygosity at distance $0 < i < k$ from the mainland we find:

$$0 = \left(-\partial_t - 1\right)F^{(i)}(t) + 2M_\mathrm{e}\left(F^{(i+1,i)}(t) + F^{(i-1,i)}(t) - F^{(i)}(t)\right) + 1 \ . \tag{S27}$$

For the island furthest from the mainland, $i = k$, the corresponding expression is:

$$0 = \left(-\partial_t - 1\right)F^{(k)}(t) + M_\mathrm{e}\left(F^{(k,k-1)}(t) - 2F^{(k)}(t)\right) + 1 \ . \tag{S28}$$

By setting the time derivatives in Eqs. (S23)-(S28) to zero, one finds the expressions for the steady-state homozygosity of the system. The steady-state heterozygosity is obtained upon subtracting the steady-state homozygosity from unity. Upon setting $H^{(0)} = 1$, the results shown in Fig. S1**D**-**F** are obtained. We note that the lines in Fig. S1**E** correspond to the lines shown in Fig. 4**B** in the main text.

References

1. Wright S (1931) Evolution in Mendelian populations. Genetics 16: 97–159.
