# Supplementary material for: The Effect of Multiple Paternity on Genetic Diversity of Small Populations during and after Colonisation
Source: PLoS One. 2013 Oct 28;8(10):e75587. doi: 10.1371/journal.pone.0075587 (PMC3810386; doi:10.1371/journal.pone.0075587)
Supplement: Appendix S4 — (PDF) [file pone.0075587.s004.pdf]

Appendix S4

This appendix discusses our method for estimating the durations of low- and high-heterozygosity phases from the results of our computer simulations, and analytically. Our scheme for computing the durations of low- and high-heterozygosity phases is shown in Fig. S2**A**. As indicated in this figure, we consider values of the heterozygosity smaller than 0.1 to be low, and values of the heterozygosity larger than 0.4 to be high (see also Section **Results** in the main text).

First, we explain the method used to record the times of transitions between low- and high-heterozygosity phases in computer simulations. Say the heterozygosity is in the high phase at time $\tau = 0$. We record first the nearest point in time when the heterozygosity becomes less than 0.1. Say this occurs in generation τ_0. Second, we search for the last generation before τ_0 in which the heterozygosity has a value larger than or equal to 0.25 (the middle point between 0.1 and 0.4). Say this happens in generation $\tau_1 < \tau_0$. We take τ_1 to be the time of transition from the high- to the low-heterozygosity phase. The transitions from the low to the high phase are recorded using a similar scheme. The resulting average durations of the high- and low-heterozygosity phase, $T_{\rm high}$ and $T_{\rm low}$, depend on the degree of multiple paternity in the population, that is on the number of available mates, s. For different values of s, the durations of high- and low-heterozygosity phases, relative to their corresponding values for $s = 1$ (computed as explained above) are shown as symbols in Fig. S2**B**, **D**, and **F**. For comparison, we show in Fig. S2**C**, **E**, and **G** the corresponding steady-state heterozygosities relative to their values for $s = 1$.

Second, we briefly explain how to estimate $T_{\rm low}$ and $T_{\rm high}$ analytically. The heterozygosity can switch from the low to the high phase if new genetic material comes to the population, and if this material is not lost due to random genetic drift. Recall that, in our model, migration is the only process bringing new genetic material to islands. However, some migrations bring genetic material that already exists in a given island population (but note that when $H^{(0)} = 1$, then the first island receives new genetic material with each migration). Yet, it is possible to estimate the effective successful migration rate per allele per generation, $m_{\rm e}^{(i)}$, at a given distance i from the mainland by using our analytical results derived for the steady-state heterozygosity in island i, namely $H^{(i)}$. Here, the term ‘successful’ implies that migrants bring new genetic material to the population. Note that under the assumption that a new allelic type is introduced to the population at distance i from the mainland at rate $m_{\rm e}^{(i)}$ per allele per generation, the steady-state heterozygosity is computed as

$$H^{(i)} = \frac{4m_{\rm e}^{(i)} N_{\rm e}}{1 + 4m_{\rm e}^{(i)} N_{\rm e}} \ , \tag{S29}$$

where $2m_{\rm e}^{(i)} N_{\rm e}$ is the total number of new allelic types introduced in the population per generation (this expression is analogous to the usual expression for the heterozygosity which involves the scaled mutation rate, see Eq. (S10) in **Appendix S1**). In the case $m_{\rm e}^{(i)} N_{\rm e} \ll 1$ it follows that typically every $(2m_{\rm e}^{(i)} N_{\rm e})^{-1}$ generations, one mother comes to an island carrying a new allele. Furthermore, it is known [1] that a large haploid population with effective size $2N_{\rm e}$ spends on average $2/N_{\rm e}$ generations in the state with this allele having the frequency $N_{\rm e}$ before fixation occurs. It follows that the typical waiting time for such a successful establishment of new genetic material in island i is equal to $(4m_{\rm e}^{(i)})^{-1}$. This estimate agrees well with the results of our computer simulations (results not shown).

We now explain how to estimate analytically the average duration of the high-heterozygosity phase, $T_{\rm high}$. In the case of rare income of new allelic types to island i, $m_{\rm e}^{(i)} N_{\rm e} \ll 1$, the island population at

a given locus typically has at most 2 alleles. Thus, T_{high} can be approximated by the time that a locus with two alleles in a Wright-Fisher population of N_{e} diploid individuals needs to reach fixation. Note that under the condition $m_{\mathrm{e}}^{(i)}N_{\mathrm{e}} \ll 1$, fixation occurs typically much before new genetic material arrives to the population. The number of generations, τ_{loss}, until fixation in a diploid population at a locus with two alleles is [2]

$$\tau_{\mathrm{loss}}(f_0) \approx -4N_{\mathrm{e}}[f_0 \ln(f_0) + (1 - f_0)\ln(1 - f_0)] \ . \tag{S30}$$

Here f_0 is the initial frequency of a given allelic type. For a given value of the number of available mates, s, we compute T_{high} upon integrating Eq. (S30) from $f_0 = 0.1$ to $f_0 = 0.9$. The integral boundaries correspond to the value of heterozygosity ≈ 0.2, which is close to the value of 0.25 in the method used for determining the transition time between the low and the high phase. We note that Eq. (S30) results in underestimated time of fixation if the rate of income of new genetic material is not too small. This is confirmed by the results shown in Fig. S2**B**, **D**, and **F**. Note that the solid lines in Fig. S2**B**, and **C** correspond to the lines in Fig. 5**B**, and **C** (respectively) in the main text.

References

1. Ewens W (1982) On the concept of the effective population size. Theoretical Population Biology 21: 373-378.

2. Hartl DL, Clark AG (1998) Principles of Population Genetics. Sinauer Associates.
